# Development and Validation of a Predictive Model for the Risk of Postoperative Frailty in Elderly Patients With Benign Brain Tumors

**DOI:** 10.1002/brb3.71429

**Published:** 2026-04-22

**Authors:** Jiahui Yu, Panpan Xu, Linjing Du, Xiuqun Xu, Yinyin Fan, Meng Sun, Yanqing Li, Xiaomei Zhang

**Affiliations:** ^1^ Department of Neurosurgery Affiliated Hospital of Nantong University Nantong China; ^2^ Department of School of Nursing and Rehabilitation Nantong University Nantong China; ^3^ Department of Nursing Affiliated Hospital of Nantong University Nantong China

**Keywords:** benign brain tumors, elderly patients, frailty, predictive model

## Abstract

**Objective:**

This study aimed to identify the key predictors of frailty in elderly patients with benign primary brain tumors (PBTs) and develop and validate a nomogram for predicting postoperative frailty in these patients, which integrates disease characteristics, individual factors, psychosocial indicators, and multidimensional health parameters.

**Methods:**

A cohort of 313 elderly patients with benign PBTs was recruited from a tertiary hospital in Nantong, China, and was then randomly split into a training set (*n* = 219) and a validation set (*n* = 94). Binary logistic regression analysis was used to identify risk factors for frailty in the training set. LASSO regression identified five key predictors, which were then used to construct a predictive model. The model's performance was evaluated in terms of discriminative ability, calibration, and clinical utility using ROC curves, calibration plots, and decision curve analysis (DCA).

**Results:**

In this study of 313 patients with benign PBTs, the five factors (KPS, Hb, MDASI‐BT, SSRS, and CD‐RISC) were identified as independent predictors of frailty in the elderly patients. In the training set as well as the validation set, the AUC was 0.936 (95% CI: 0.90–0.97) and 0.939 (95% CI: 0.89–0.98). DCA further verified the favorable predictive efficacy of the model.

**Conclusion:**

We developed a reliable predictive model to predict frailty in elderly patients with benign PBTs after undergoing intracranial tumor resection. This model is intended to help clinical staff assess frailty risk and screen high‐risk patients.

## Graphical abstract

This study developed and validated a nomogram for predicting postoperative frailty risk in elderly patients with benign brain tumors. The model incorporates five key predictors and serves as a practical tool for preoperative risk assessment and stratification.

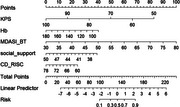

## 1 Background

Benign primary brain tumors (PBTs) are nonmalignant intracranial tumors characterized by abnormal cell growth in or near the brain. The 2022 World Health Organization (WHO) report indicates that the global number of new brain tumor cases was 321,476, accounting for 1.6% of all cancers (Bray et al. 2024). The 5‐year survival rate of patients with a benign PBT is 91.8% (Ostrom et al. 2023). While these tumors may be histologically benign, they are capable of causing mass effects and compressing vital structures like the optic nerve, language areas, or the pituitary gland, resulting in abnormal findings on imaging or clinical examination. Even small‐sized tumors may lead to visual loss (Tang et al. 2023) and multiple endocrine deficits. In an emergency situation, a patient may face life‐threatening complications due to the mass effect of the tumor. Since benign brain tumors grow slowly and symptoms develop gradually, they are often misdiagnosed as “migraines,” “common aging,” or other chronic conditions, and treatment is delayed. When a structure remains compressed for a longer period, irreversible damage may occur, and the recovery may be incomplete even after tumor excision.

In the context of global population aging, the health of the elderly has become an increasingly important public health problem (Yanan et al. 2021). Elderly patients usually have decreased physiological reserve, reduced function of multiple organs, and decreased metabolic capacity, which often coexist with more severe tumor mass effects and a lower quality of life (Li and Ma 2024). These inherent physiological deficits are also likely to predispose elderly patients with neurological disorders to multidimensional functional impairment subsequent to disease onset (Y. Liu et al. 2024). Although surgery is still the first‐line treatment for this disorder (Avila et al. 2024), elderly patients frequently have a reduced capacity to undergo surgeries due to the higher prevalence of age‐related conditions. While resection for benign brain tumors is relatively safe, postoperative frailty is a common condition, and it often parallels the process of rehabilitation. As the eighth most prevalent tumor in the country, brain tumors show peak numbers of cases and deaths in adults aged 60 years and older, and the incidence in the elderly population is increasing annually (Hou et al. 2022). Thus, older adults with benign PBTs represent a sizeable patient group that frequently experiences symptoms related to frailty. Existing clinical guidelines are largely based on data from younger populations (Mungngam et al. 2022; She et al. 2024) and lack individualized strategies for elderly patients, who have limited compensatory capacity and for whom early intervention is particularly important.

Frailty is an age‐related decline in physiological reserve and is commonly manifested by weight loss, fatigue, and reduced muscle strength. Frailty in elderly individuals is linked to an increased risk of unfavorable health outcomes, such as falls (Nisar et al. 2024), postoperative delirium (Bellelli et al. 2024), and disability (Galluzzo et al. 2023). This condition is often associated with a diminished reserve of balance and reduced or absent skeletal muscle strength. Tumor compression has been associated with gait disturbances and an increased risk of hip fractures in elderly patients, the latter of which can be life‐threatening. Many struggle to independently carry out regular daily routines, typically as a result of reduced muscle strength or impaired balance, which can lead to restricted independent mobility. Furthermore, the management of benign tumors typically emphasizes functional preservation, given the potential for long‐term survival. Frailty is often accompanied by increased dependence on others for care, which may coincide with a more rapid progression of disability, withdrawal from social activities, deterioration of social functioning (Fang et al. 2022), and the presence of emotional problems or suicidal tendencies (Y. Yang et al. 2024). Frailty in patients with benign brain tumors is often observed alongside poorer outcomes, potentially in the context of further declines in cardiorespiratory function and immune response, which may coexist with multisystem complications (Jimenez et al. 2023). These complications may, in turn, be associated with slower recovery, greater physical depletion, and increased risks of mortality and disability. By assessing mechanisms related to frailty, potential intervention targets may be identified to help prevent complications. Therefore, identifying elderly patients with benign brain tumors who are at risk of frailty following tumor resection is crucial.

Nomograms, as visual predictive tools, are widely used in clinical and non‐clinical studies and provide good predictive performance. By integrating multidimensional variables, they can quantify individual risk and support clinical decision‐making and have demonstrated value in the assessment of tumor prognosis. However, in neuro‐oncology, the application of nomograms has mainly focused on survival outcomes in malignant tumors (C. Yang et al. 2025; Smith et al. 2025) or on younger age groups (Huq et al. 2021; Yuan et al. 2023), with limited data specific to elderly patients with benign tumors. These patients face the combined influences of aging and brain tumor‐related functional impairment. Although survival is often prolonged, frailty may coexist with long‐term cognitive impairment and an increased risk of falls. A predictive model for assessing postoperative frailty among elderly individuals with benign brain tumors could provide a basis for preoperative assessment and postoperative rehabilitation resource allocation. This study constructed a nomogram emphasizing the significance of disease‐specific characteristics in frailty assessment and helping clinicians recognize the individuals at high risk of frailty at the time of preoperative evaluation, treatment decision, and rehabilitation planning to facilitate more rational resource allocation and individual intervention strategies.

This study aimed to construct and validate a predictive model for assessing the risk of postoperative frailty at discharge in patients undergoing craniotomy for intracranial tumors. In this study, postoperative frailty was defined as the presence of unexplained functional decline after surgery, requiring assistance from others to perform activities of daily living at discharge, along with a Tilburg Frailty Indicator (TFI) score of ≥5 on the day of discharge. Hospital discharge does not equate to full recovery. By enabling early identification of frailty risk and informing timely interventions, this approach may support better reintegration into daily life and contribute to improved long‐term quality of life.

## 2 Methods

### 2.1 Study Design and Participants

This was an observational prospective study. A total of 313 elderly patients with benign brain tumors were recruited between January 2023 and March 2025 at a tertiary care hospital in Nantong, China, using a convenience sampling approach. Eligibility criteria included the following: (1) aged ≥60 years, with histopathologically confirmed benign PBTs based on the WHO classification of central nervous system tumors; (2) patients who were assessed with the TFI at admission and confirmed to be non‐frail, defined as a TFI score <5; (3) absence of severe cognitive impairment or psychiatric disorders; (4) voluntary participation and signed informed consent; and (5) sufficient communication ability without language barriers. Exclusion criteria were as follows: (1) frailty at admission, defined as a TFI score ≥5 and (2) poor compliance or inability to complete the study‐related assessments.

### 2.2 Measurement Tools

#### 2.2.1 Demographic and Clinical Characteristics

General information included gender; age; body mass index; work status; income status; education level; living conditions; consumption of fruits, vegetables, alcohol, and tobacco; exercise habits; financial stress; Karnofsky Performance Status (KPS); nutritional status; and chronic disease coexistence. Clinical data included biochemical indicators (including hemoglobin, blood urea nitrogen, red blood cell count, etc.), intraoperative blood loss, duration of surgery, and postoperative complications.

#### 2.2.2 The Frailty Scale

Gobbens et al. (2010) created the TFI in 2010. The 15 items on the TFI are divided into three categories: social, psychological, and physical. A score of ≥5 indicates the existence of frailty. Scores range from 0 to 15.

#### 2.2.3 Mini‐Nutritional Assessment Short Form

The Mini‐Nutritional Assessment (MNA), originally developed by Rubenstein et al. (2001), was further streamlined and utilized as the questionnaire. Higher scores indicate better nutritional status. This tool comprises six items, with total scores ranging from 0 to 14. This scale has a Cronbach's *α* coefficient of 0.843.

#### 2.2.4 M.D. Anderson Symptom Inventory Brain Tumor Module

Developed by Terri et al. (2005) within the M. D. Anderson Cancer Center, this scale is designed to evaluate symptom severity and the impact on daily life in patients with brain tumors. It comprises two sections with 28 items; higher total scores correspond to greater symptom severity and interference. The scale demonstrates acceptable reliability, with a Cronbach's *α* coefficient of 0.762.

#### 2.2.5 Pittsburgh Sleep Quality Index

The Pittsburgh Sleep Quality Index (PSQI), created by Buysse et al. (1989) in 1993, is the most commonly used sleep quality assessment scale. It comprises 15 items across seven dimensions, with total scores ranging from 0 to 21; lower scores indicate better sleep quality. For the present sample, the Cronbach's α coefficient was calculated to be 0.713.

#### 2.2.6 The Connor‐Davidson Resilience Scale

This scale comprises 25 items across three dimensions—tenacity, resilience, and optimism—was first created by American psychologist Connor and Davidson (2003). Greater resilience is indicated by higher total scores, which range from 0 to 100. The scale in this study has a Cronbach's *α* coefficient of 0.889.

#### 2.2.7 Brief Ageing Perceptions Questionnaire

Based on the original Ageing Perceptions Questionnaire (APQ), Na et al. (2018) redesigned this measure. A 5‐point Likert scale is used, with responses ranging from 1 to 5. Higher overall scores on the measure, which has 17 items and five aspects, indicate that older people have more unfavorable attitudes about aging. The Cronbach's *α* coefficient for this study was 0.77.

#### 2.2.8 Fear of Progression Questionnaire‐Short Form

It is a shortened version of the original scale developed by Mehnert et al. (2006), based on the work of Zimmermann et al. (2011). In 2015, Qiyun et al. (2015) developed a Chinese version of the Fear of Progression Questionnaire‐Short Form. It comprises 12 items and assesses physical health and social and family functioning. The scale uses 5‐point Likert scale. The instrument demonstrates favorable internal consistency, with a Cronbach's *α* coefficient of 0.82.

#### 2.2.9 Social Support Rating Scale

The scale, created by Shuiyuan and Yang (1987), was used to measure social support. Ten items make up the scale, which evaluates social support in three areas. An individual who receives more social support has a higher overall score on the scale. The scale has a Cronbach's *α* coefficient of 0.754.

### 2.3 Data Collection Procedures

This study selected patients with benign PBTs admitted to the neurosurgery department of a tertiary hospital in Nantong as the research subjects. All potential participants underwent frailty assessment using the TFI at admission. This study included only participants who showed no signs of frailty at admission (TFI score <5). The final sample consisted of 313 participants who were eligible for enrollment. Study questionnaires were completed by all participants before hospital discharge. Prior to distribution, researchers explained the relevance and implications of this research for patients with benign PBTs and obtained their consent. All participants took part voluntarily, signed written informed consent forms, and were explicitly informed that all research data would be kept confidential. The collected data encompassed sociodemographic characteristics, preoperative, intraoperative, and postoperative clinical data, alongside admission assessments of all scales. Frailty status was re‐evaluated on the day of hospital discharge. When participants had questions about the questionnaire, standardized explanations were provided. Completed questionnaires were collected on site by trained members of the research team.

### 2.4 Ethics Statement

The Ethics Committee at Nantong University Hospital approved the study, which adhered to the Declaration of Helsinki (2022‐L081).

### 2.5 Statistical Analyses

The assembled dataset was randomly partitioned into training and validation subsets at a pre‐specified 7:3 ratio. Missing data were managed via mean imputation, with missingness rates documented as follows: physical activity (1.37%), lactate dehydrogenase (LDH, 0.91%), and calcium (Ca, 0.91%). Variables that follow a normal distribution are shown as mean ± standard deviation (SD), whereas data not normally distributed are represented as median (M) with interquartile range (IQR; P_25_–P_75_). Inter‐group comparisons were analyzed using independent sample *t*‐tests and Mann–Whitney *U* tests, respectively. To evaluate differences between groups in categorical variables, which were shown as frequencies and percentages, the Pearson chi‐square test and Mann–Whitney *U* test were employed.

This study adopted binary logistic regression analysis for the training dataset, aiming to identify the separate risk factors associated with frailty. Statistical significance was defined as a two‐sided *p* < 0.05. This model yielded a predicted probability *P*(*X*) ranging from 0 to 1, which was derived via the inverse logit transformation to quantify the probability of developing frailty in elderly individuals diagnosed with benign PBTs who underwent intracranial tumor resection surgery:

(1) $$P(X)=\frac{1}{1+e^{-\mathrm{LP}}},$$

where LP represents the multivariable linear predictor, which is calculated using the following equation:

(2) $$\mathrm{LP}=\beta_{0}+\beta_{1}X_{1}+\cdots +\beta_{i}X_{i},$$

where *β*_0_ is the y‐intercept and *β*_1_…*β*_i_ is the respective slope/coefficient of the given candidate predictor, *X*_1_…*X*_i_. Significant factors influencing postoperative frailty in elderly patients with benign PBTs were found using least absolute shrinkage and selection operator (LASSO) binomial regression analysis. LASSO regression enables feature selection and ensures that there is no overfitting through the introduction of an L1 regularization penalty term. The efficacy of the classification model was assessed via the area under the ROC curve and visualized by plotting calibration plots using a 1000 bootstrap resampling procedure (Y. Liu et al. 2024). Finally, decision curve analysis was used to assess the value of decision curve analysis (DCA) for predictive modeling in practical clinical decision‐making.

## 3 Results

### 3.1 Baseline Clinical Characteristics

Of the 313 patients with benign PBTs included in this study, 141 (45.0%) were males and 172 (55.0%) were females, 119 (38.0%) were patients with pituitary tumors, 152 (48.6%) were patients with meningiomas, 34 (10.9%) were patients with benign tumors of the auditory nerves, and 8 (2.9%) were patients with other benign brain tumors. Detailed characteristics are shown in Table 1. Upon comparison, no statistically significant intergroup differences were observed with respect to age, gender, economic pressure, educational level, KPS score, and hemoglobin (Hb) concentration (*p* > 0.05).

**TABLE 1:** Features of the participants in the training cohort and the validation cohort (*n* = 313).

Variables	Training cohort (*n* = 219)	Validation cohort (*n* = 94)	All	*p* value
Age (years)	68 (64, 73)	67.5 (63, 71)	68 (64, 72)	0.429
Gender, n (%)				0.569
Male	103 (47.0)	38 (40.4)	141 (45.0)	
Female	116 (53.0)	56 (59.6)	172 (55.0)	
BMI	24.51 ± 3.14	23.97 ± 2.85	24.35 ± 3.06	0.152
Work status, n (%)				0.332
Incumbency	67 (30.6)	22 (23.4)	89 (28.4)	
Retire	137 (62.6)	67 (71.3)	204 (65.2)	
Unemployed	15 (6.8)	5 (5.3)	20 (6.4)	
Income (CNY), n (%)				0.168
<3000	123 (56.2)	62 (66.0)	185 (59.1)	
3000–6000	80 (36.5)	24 (25.5)	104 (33.2)	
>6000	16 (7.3)	8 (8.5)	24 (7.7)	
Education, n (%)				0.740
University and above	5 (2.3)	1 (1.1)	6 (1.9)	
High school or junior college	42 (19.2)	17 (18.1)	59 (18.8)	
Junior high school or vocational school	72 (32.9)	36 (38.3)	108 (34.5)	
Elementary school and below	100 (45.7)	40 (42.6)	140 (44.7)	
Living conditions, n (%)				0.992
Spouse	170 (77.6)	73 (77.7)	243 (77.6)	
Living alone	10 (4.6)	4 (4.3)	14 (4.5)	
Children	39 (17.8)	17 (18.1)	56 (17.9)	
Smoking, n (%)				0.718
Yes	19 (8.7)	7 (7.4)	26 (8.3)	
No	200 (91.3)	87 (92.6)	287 (91.7)	
Drinking, n (%)				0.228
Yes	48 (21.9)	15 (16.0)	63 (20.1)	
No	171 (78.1)	79 (84.0)	250 (79.9)	
Daily intake of fruit, n (%)				0.908
Yes	87 (39.7)	38 (40.4)	125 (39.9)	
No	132 (60.3)	56 (59.6)	188 (60.1)	
Daily intake of vegetables, n (%)				0.548
Yes	206 (94.1)	90 (95.7)	296 (94.6)	
No	13 (5.9)	4 (4.3)	17 (5.4)	
Exercise, n (%)				0.399
Never	35 (16)	21 (22.3)	56 (17.9)	
Infrequent	108 (49.3)	42 (44.7)	150 (47.9)	
Regularity	76 (34.7)	31 (33.0)	107 (34.2)	
Economic pressure, n (%)				0.144
None	54 (24.7)	31 (33.0)	85 (27.2)	
Lightly	115 (52.5)	45 (47.9)	160 (51.1)	
Moderately	46 (21.0)	18 (19.1)	64 (20.4)	
Severely	4 (1.8)	0	4 (1.3)	
Diagnose, n (%)				0.948
Pituitary tumor	83 (37.9)	36 (38.3)	119 (38.0)	
Meningioma	105 (47.9)	47 (50.0)	152 (48.6)	
Acoustic neurinoma	25 (11.4)	9 (9.6)	34 (10.9)	
Other	6 (2.7)	2 (2.1)	8 (2.6)	
KPS	80 (80, 90)	80 (70, 90)	80 (75, 90)	0.487
Hb	128.08 ± 14.08	125.61 ± 13.80	127.34 ± 14.02	0.153
WBC	5.8 (4.8, 6.9)	5.35 (4.2, 6.68)	5.64 (4.61, 6.9)	0.045
RBC	4.19 ± 0.45	4.11 ± 0.42	4.17±0.44	0.135
BUN	5.8 (4.9, 7.2)	6.05 (5.2, 7.43)	5.9 (4.9, 7.3)	0.440
UA	322.63 ± 90.85	340.09 ± 93.58	327.87 ± 91.88	0.124
Cr	60 (49, 71)	59.5 (51.75, 69.25)	60 (50, 71)	0.889
Alb	38.6 (36.8, 41)	39.25 (37.28, 41.8)	38.8 (37, 41.05)	0.053
PA	252 (223, 280)	254 (213.5, 291)	254 (223, 284)	0.544
LDH	193 (168, 218)	190.5 (168.5, 218.25)	193 (168, 218)	0.928
Na	141 (140, 142)	141.5 (140, 143)	141 (140, 142)	0.156
Ca	2.23 (2.16, 2.28)	2.24 (2.18, 2.33)	2.23 (2.16, 2.30)	0.107
K	3.81 ± 0.40	3.74 ± 0.41	3.79 ± 0.40	0.132
MNA‐SF	13 (12, 14)	13.5 (13, 14)	13 (12, 14)	0.660
MDASI‐BT	44 (38, 60)	50 (44.75, 55)	48 (39, 56.5)	0.037
SSRS	44 (41, 47)	45 (44, 48)	45 (42, 47)	<0.001
PSQI	7 (5, 9)	8 (6, 9)	7 (6, 9)	<0.001
CD‐RISC	67 (66, 70)	65.5 (63, 67)	67 (65, 69)	<0.001
SPA	50 (45, 55)	50 (47, 54)	50 (46, 55)	0.445
FOP	30 (28, 34)	32 (30, 34)	31 (29, 34)	0.003

Abbreviations: CD‐RISC, Connor‐Davidson Resilience Scale; FOP, Fear of Progression; MNA‐SF, Mini‐nutritional assessment short‐form; MDASI‐BT, M.D. Anderson Symptom Inventory Brain Tumour Module; PSQI, Pittsburgh Sleep Quality Index; SPA, Self‐Perceptions of Aging; SSRS, Social Support Rating Scale.

The values separated by commas in parentheses represent the median, 25th percentile, and 75th percentile of each variable, respectively, which are used to describe the central tendency and distribution of the data.

### 3.2 Incidence of Postoperative Frailty

Of the 219 participants included in the training cohort, 84 cases (38.4%) showed frailty at discharge, while 135 cases (61.6%) did not. Of the 94 individuals enrolled in the validation cohort, 35 cases (37.2%) had frailty at discharge, and 59 cases (62.8%) did not. A total of 119 cases (38.0%) in both groups showed frailty.

### 3.3 Development of Predictive Models

**FIGURE 1:**
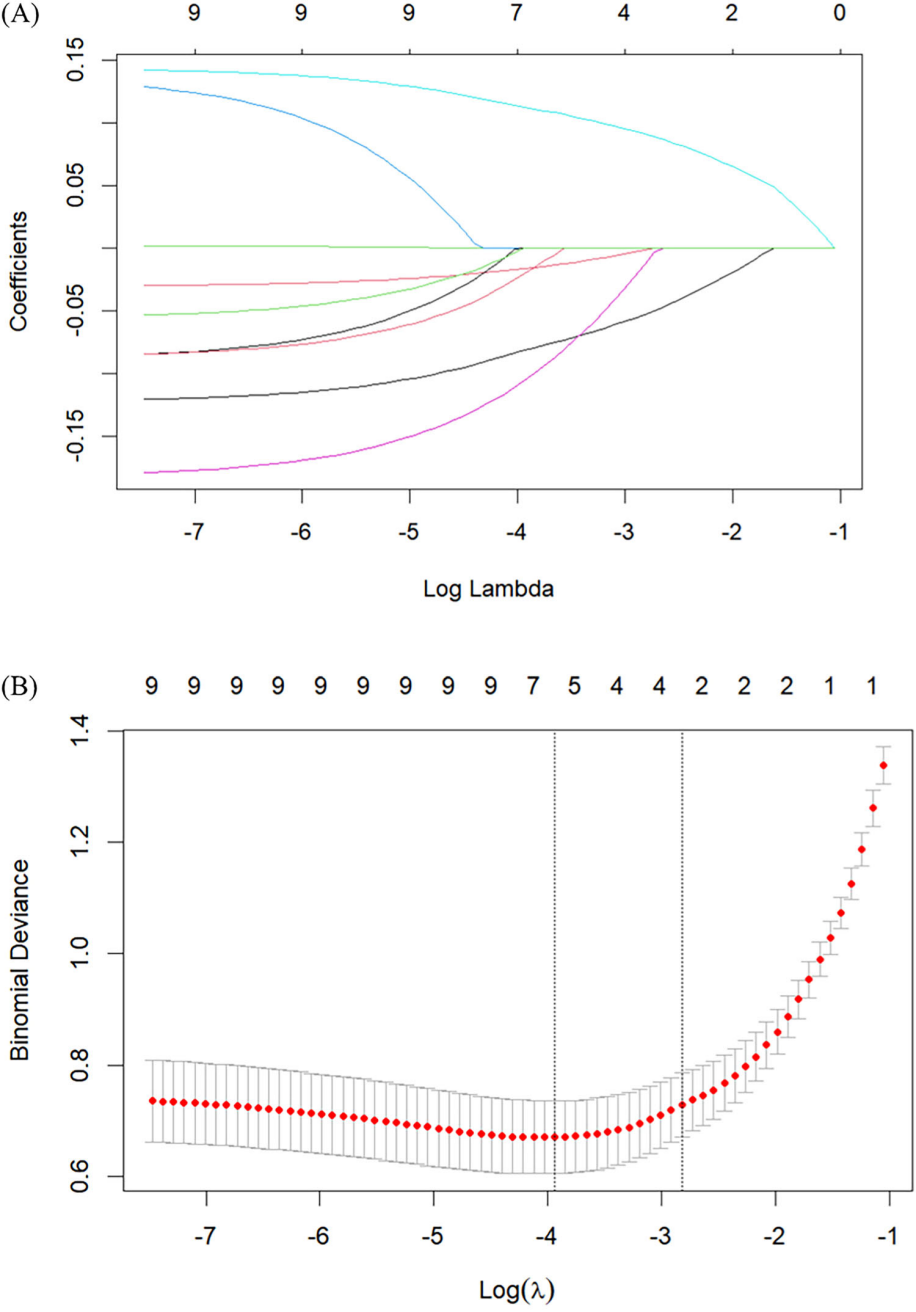
(A) The LASSO binomial regression model was used to select clinical features. A sequence of log λ (lambda) is used to construct the distribution of coefficients, and the optimal lambda generates non‐zero coefficients. (B) Ten‐fold cross‐validation with the minimum criterion was used to determine the LASSO model's optimal parameter (lambda). The minimal value is used to plot the virtual vertical line at the optimum.

**FIGURE 2:**
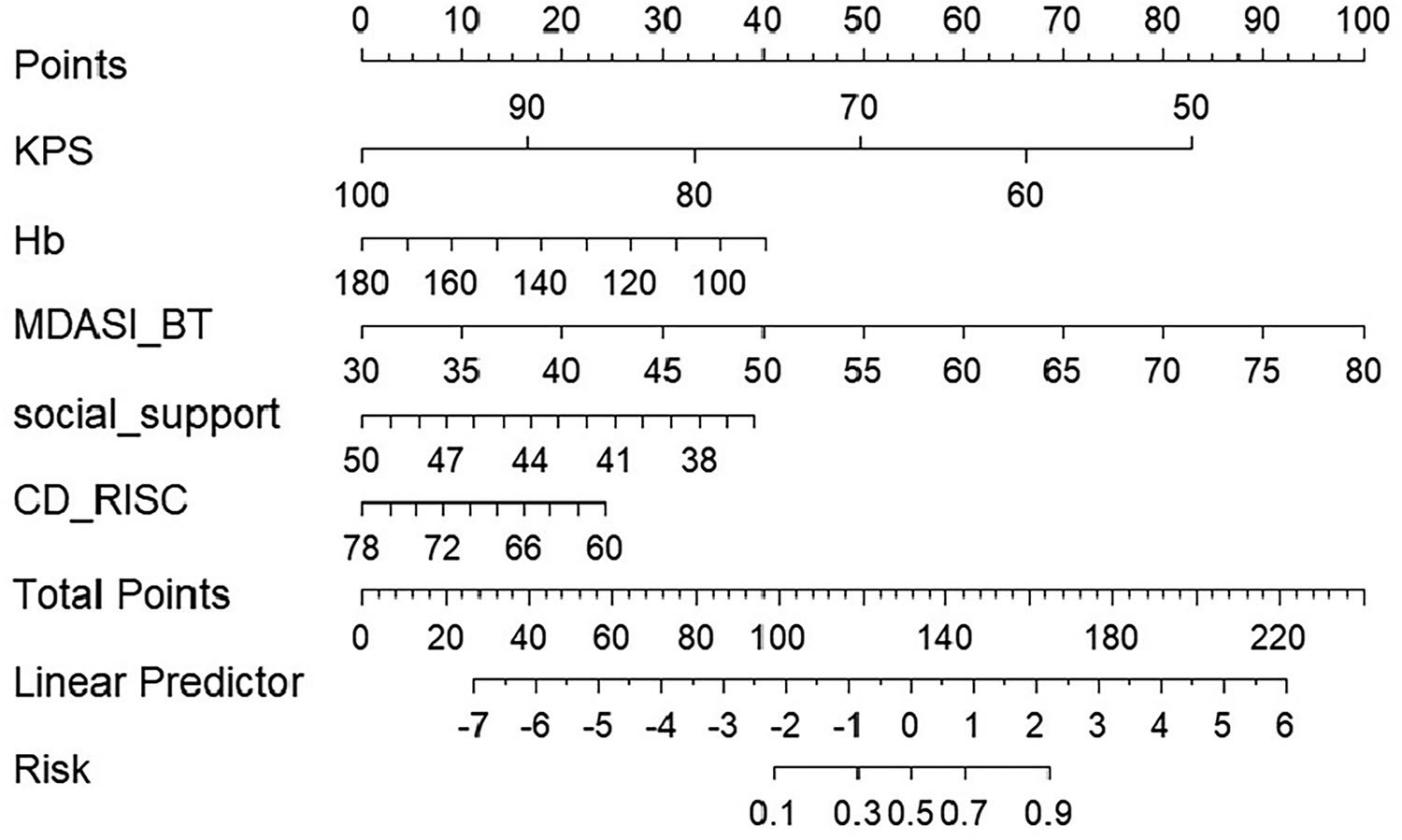
Nomogram forecasting the likelihood of frailty.

We performed univariate binary logistic regression analyses as shown in Table 2. Factors significantly associated with frailty in univariate analysis included age, KPS, Hb, LDH, Mini‐Nutritional Assessment Short Form, M.D. Anderson Symptom Inventory Brain Tumor Module (MDASI‐BT), Social Support Rating Scale (SSRS), PSQI, Connor‐Davidson Resilience Scale (CD‐RISC), self‐perceptions of aging, and fear of progression. Variables with a significant association with frailty (*p* < 0.05) were included in LASSO regression analysis. LASSO regression identified potential predictors (Figure 1A), with five key variables confirmed as independent predictors in the final analysis (Figure 1B). The probability of postoperative frailty in the study population can be calculated through the equation where LP = 19.97938 + (–0.11053) × KPS + (–0.02986) × Hb + 0.1334 × MDASI‐BT + (–0.18688) × SSRS + (–0.08955) × CD‐RISC. Based on the findings from logistic regression analysis, a nomogram model for predicting frailty risk was developed using R Studio (version 4.4.1). The final model incorporated five variables: KPS score, Hb level, MDASI‐BT score, SSRS score, and CD‐RISC score, as shown in Figure 2.

**TABLE 2:** Univariate logistic regression analysis affecting frailty in elderly patients with brain tumors.

Variables	*β*	Standard error	Wald value	*p* value	OR
Age	0.077	0.025	9.913	0.002	1.08
KPS	−0.178	0.025	50.379	<0.001	0.837
Hb	−0.055	0.012	20.729	<0.001	0.947
LDH	0.006	0.003	4.122	0.042	1.006
MNA‐SF	−0.263	0.12	4.827	0.028	0.769
MDASI‐BT	0.163	0.02	66.995	<0.001	1.176
SSRS	−0.111	0.041	7.304	0.007	0.895
PSQI	0.176	0.058	9.261	0.002	1.192
CD‐RISC	−0.108	0.041	6.937	0.008	0.898
SPA	0.096	0.024	15.718	<0.001	1.10
FOP	0.115	0.033	12.266	<0.001	1.122

Abbreviations: CD‐RISC, Connor‐Davidson Resilience Scale; FOP, Fear of Progression; Hb, hemoglobin; LDH, lactate dehydrogenase; KPS, Karnofsky Performance Status; MNA‐SF, Mini‐nutritional assessment short‐form; MDASI‐BT, M.D. Anderson Symptom Inventory Brain Tumour Module; PSQI, Pittsburgh Sleep Quality Index; SPA, Self‐Perceptions of Aging; SSRS, Social Support Rating Scale.

### 3.4 Validity and Internal Validation of Predictive Models

**FIGURE 3:**
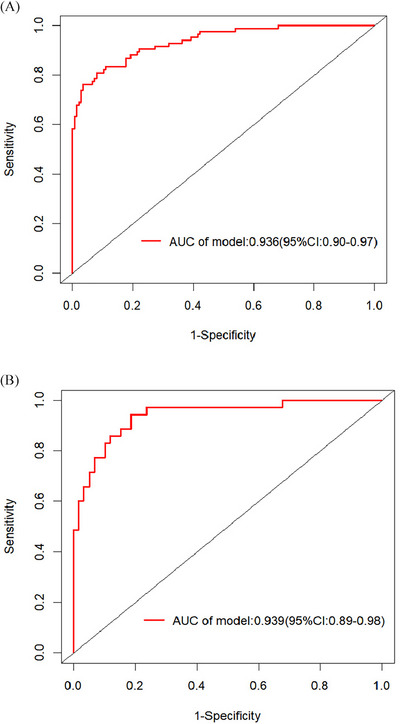
ROC curves for frailty risk prediction models. (A) Training set. (B) Validation set. AUC: area under the curve.

**FIGURE 4:**
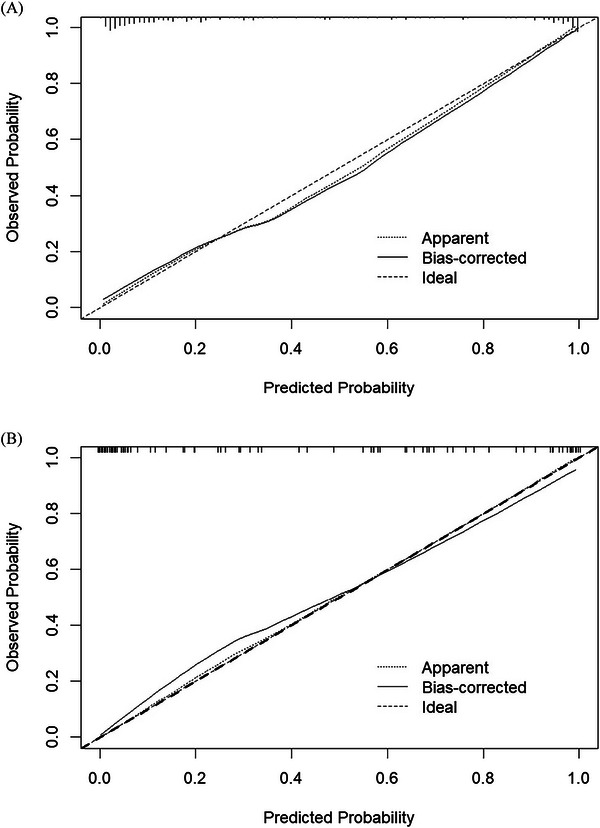
Calibration curve for the nomogram. (A) Training set. (B) Validation set.

**FIGURE 5:**
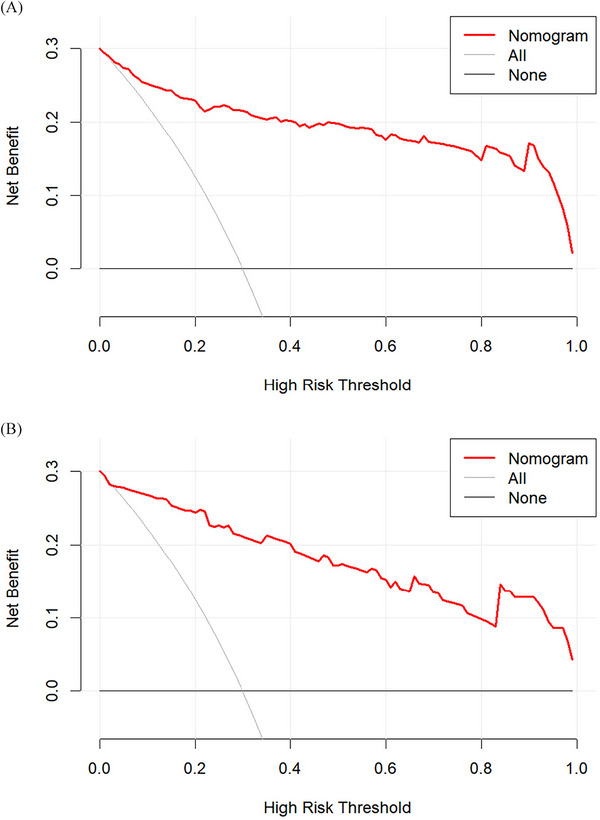
The DCA curve of the nomogram prediction model. (A) Training set. (B) Validation set.

ROC curve analysis combined with the Hosmer–Lemeshow test was applied to assess the model's predictive performance, with *p* > 0.05 representing an acceptable fitting effect. The statistical results demonstrated satisfactory goodness of fit in both the training cohort (*χ* ^2^ = 3.5611, *df* = 8, *p* = 0.8944) and the validation cohort (*χ* ^2^ = 3.7998, *df* = 8, *p* = 0.8747). ROC curve analysis was further applied to evaluate the model's ability to identify frailty among patients with benign PBTs. The area under the ROC curve was calculated to quantify discriminative capacity, with values of 0.936 (95% CI: 0.90–0.97) and 0.939 (95% CI: 0.89–0.98) in the training and validation sets, respectively (Figure 3). The bootstrap resampling method with 1000 repetitions was used for internal validation. Calibration curves of the established nomogram (Figure 4) revealed strong concordance between the predicted likelihood and the observed incidence. DCA was conducted to evaluate the clinical utility of the established model (Figure 5), indicating the nomogram's favorable clinical net benefit and predictive accuracy.

## 4 Discussion

In the current analysis, the rate of frailty among individuals was 38%, which is higher than the 30.1% reported by Dicpinigaitis et al. (2021) in patients with intracranial meningiomas. This difference may be related to variations in the age composition of the study populations. The participants in the present study were elderly, a group with a higher prevalence of frailty than the general adult population, partly due to chronic illnesses (Xu et al. 2024) and loss of muscle mass. In addition, elderly individuals are more likely to experience loneliness, often accompanied by psychological distress. These psychological factors frequently coexist with, or are associated with, frailty (X. Liu et al. 2023).

In this study, the relationship between frailty and age was examined using a multidimensional approach, and frailty was not necessarily associated with advancing age alone. Although frailty is commonly regarded as a manifestation of the aging process (Zeng et al. 2023), the findings suggest that its onset and progression may involve substantial contributions from non‐age‐related factors. This observation is consistent with the possibility that core features of frailty are reflected in specific biochemical indicators (Schwartz et al. 2024). In addition, frailty progression may not follow a linear pattern with age. Future studies could employ machine learning models, such as random forests, to explore the complex interactions among these variables. These findings challenge the assumption that frailty is an inevitable consequence of aging and call for a comprehensive approach to address its multifactorial mechanisms rather than focusing solely on age. Further research may help clarify the key drivers of frailty to support individualized intervention strategies.

The present study found that a lower KPS score was significantly correlated with frailty. KPS assesses overall functional status and quality of life by estimating the patient's extent of self‐reliance in completing basic daily functional activities and self‐care. Given that frailty is characterized by diminished physiological reserve and functional capacity, patients with a low KPS score have sarcopenia, fatigue, and difficulty walking, which are clinical symptoms of frailty. Frailty coexists with systemic low‐grade inflammation (Baechle et al. 2023) and metabolic disorders (Choi et al. 2024), including sarcopenia. Patients with poor functional status tend to have high levels of inflammatory markers (Wu et al. 2023), which contribute to the progression of frailty. The present findings indicate that it is appropriate to include the KPS in the early assessment of frailty based on clinical judgment. In contrast to complex assessment tools, the KPS can be easily evaluated and used in busy clinical practice settings.

While previous studies have reported (Lee et al. 2021) an association of lower Hb with risk of sarcopenia, mobility disability, and frailty, the stand‐alone contribution of Hb in predicting frailty risk shown in this study indicates its significance as a potential marker of frailty. Lower Hb levels result in reduced oxygen delivery to tissues leading to impaired energy metabolism and diminished capacity for muscle repair, often preceding muscle mass and strength loss. Lower Hb levels also often coexist with chronic inflammation, raised IL‐6, C‐Reactive Protein (CRP), ferritin abnormalities, which are observed in conditions such as anemia and muscle catabolism (Manzoor et al. 2023). It suggests that there could be a pathophysiologic pathway through which anemia and inflammation may be associated with frailty. As Hb is a routinely examined parameter, it can be easily incorporated into the assessment of frailty risk. The awareness of the risk of frailty with lower Hb levels may also arise in clinical practice and necessitate interventions like treating anemia, providing appropriate nutrition, close monitoring of muscle function, and so forth. The relationship between changes in Hb over time and progression or improvement of frailty needs further exploration.

MDASI‐BT scores were found to be an independent predictor of postoperative frailty. This interesting result suggests that symptom burden, such as pain, fatigue, and neurocognitive symptoms, could also contribute to the onset of frailty beyond the direct effects of the tumor itself or its treatment. Pain, fatigue, and neurologic symptoms as assessed by the MDASI‐BT may lead to reduced levels of physical activity. Reduced physical activity may contribute to muscle wasting, reduced mobility, inability to perform daily living tasks independently, reduced treatment adherence, and poor self‐care (Quach et al. 2023), all promoting the development of frailty through a behavioral pathway (Brady and Cohen 2023). Symptom control appears to be an important aspect of frailty prevention and intervention in patients with benign PBTs. The incorporation of the MDASI‐BT in regular evaluations of patients with benign PBTs may help identify those patients with high symptom burden and those at increased risk for developing frailty. Pharmacologic and non‐pharmacologic strategies for symptom management (pain control, anti‐fatigue medications, cognitive rehabilitation) and tailored exercise programs should be considered in patients with high MDASI‐BT scores.

Social support was a significant predictor of frailty, which is consistent with the finding of Yao et al. (2025). In addition to having greater health‐related needs than non‐cancer patients, elderly patients with brain tumors may perceive themselves as a burden to their family. This perception may make them more reluctant to ask for help and support from others and may lead to a sense of worthlessness. Strong social support from others is linked to better access to medical care, personal care, and emotional support and companionship in daily living, factors which are associated with slower functional decline. In caring for an elderly patient with benign PBTs, the promotion of health care provider and family caregiver engagement can reduce a sense of hopelessness. It can also provide opportunities to form new social roles and to feel that one's connections with others are meaningful.

The CD‐RISC score was also predictive. High psychological resilience means being more capable of coping with stress, or experiencing slower physiological decline because of adaptive coping strategies such as physical exercise, seeking help proactively, and preserving healthy behaviors (Wang et al. 2024). The score could be associated with interpreting declining function in a positive way, indirectly supporting motivation for recovery and slow disability progression through enhanced self‐efficacy and optimism (Zhou et al. 2024). The independent association between the CD‐RISC scores and frailty may indicate a protective role of psychological resilience in the processes of frailty and can be viewed as supporting inclusion of psychosocial factors in a broad biopsychosocial framework of frailty. Our findings suggest clinical benefits in incorporating approaches beyond those focused solely on biochemical treatments to incorporate attention to the interrelated role of psychological and physiological therapies.

A frailty predictive nomogram for elderly patients with benign PBTs can systematically incorporate biological, symptom‑related, and psychosocial factors, enabling a more comprehensive evaluation of the multidimensional nature of frailty. The construction of a frailty map would not only provide theoretical insights into the complex and multi‐domain features of frailty but would also be translated into a practical tool that can guide clinical decision‐making.

Several limitations should be acknowledged in this study. (1) The inclusion of predominantly inpatients who underwent surgical treatment may have excluded patients with milder conditions who did not receive surgery or were managed through outpatient follow‐up. As a result, the predictive performance of the model for non‐surgical patients remains unverified. (2) Heterogeneity may exist in frailty‐related factors among benign brain tumors with different pathological types, such as meningiomas and pituitary tumors; however, subgroup analyses were not conducted in this study. Despite internal validation using the bootstrap method, the absence of external validation with independent cohorts limits the generalizability of the model. In addition, the limited sample volume and single‑institution study design may compromise the stability and generalizability of the model and do not fully exclude the possibility of overfitting. (3) As this study relied solely on the TFI to assess frailty and did not include comparative analyses with established models, such as the Fried phenotype or the CFS, future studies should incorporate multiple assessment tools for direct comparison. (4) Cultural differences in the interpretation of scale items may also affect generalizability. Future research should seek validation through multicenter and cross‐cultural studies and consider the use of more adaptable assessment instruments.

## 5 Conclusion

We used LASSO regression for variable selection to identify the top predictors of frailty in elderly patients with benign PBTs and developed a nomogram to predict the risk of frailty in these patients. This study identified KPS, Hb, MDASI‐BT, SSRS, and CD‐RISC as the optimal predictors of frailty in elderly patients with benign PBTs undergoing intracranial tumor resection. These findings hold significant clinical implications for developing predictive strategies and optimizing frailty management in this specific patient population.

## Author Contributions

**Jiahui Yu**: conceptualization; writing – original draft; validation. **Panpan Xu**: writing – review & editing; data curation; visualization. **Linjing Du**: formal analysis; methodology. **Xiuqun Xu**: conceptualization. **Yinyin Fan**: data curation. **Meng Sun**: visualization. **Yanqing Li**: investigation. **Xiaomei Zhang**: project administration; funding acquisition; software; supervision; resources.

## Funding

This work was supported by the Nantong Social and People's Livelihood Science and Technology Plan under Grant number MS2024060 and the Science and Technology Department of the Affiliated Hospital of Nantong University (Grant No. Tfh2603).

## Ethics Statement

Before completing the questionnaire, the purpose of the study was explained to the participants by the researchers, and informed consent was obtained. This study was performed in line with the principles of the Declaration of Helsinki and approved by the Ethics Committee of Nantong University Hospital (2022‐L081).

## Conflicts of Interest

The authors declare no conflicts of interest.

## Data Availability

The data analyzed in this study cannot be publicly shared due to privacy protection reasons.
